# Crystal structure of tri­benzyl­bis­(tetra­hydro­furan-κ*O*)lutetium(III)

**DOI:** 10.1107/S2056989017018254

**Published:** 2018-01-09

**Authors:** Kuburat O. Saliu, Josef Takats, Robert McDonald

**Affiliations:** a Department of Chemistry, University of Alberta, Edmonton, AB T6G 2G2, Canada

**Keywords:** lanthanide, lutetium, penta­coordinate, benz­yl, crystal structure

## Abstract

In the title compound, [Lu(C_7_H_7_)_3_(C_4_H_8_O)_2_] (**1**), the Lu ion is coordinated by three benzyl and two tetra­hydro­furan ligands. Two of the benzyl groups are bonded in a classical η^1^-fashion through the methyl­ene *via* the *ipso*-carbon atom of the benzyl ligand in addition to bonding through the methyl­ene C atom, resulting in a modified trigonal–bipyramidal coordination geometry about the Lu center.

## Abstract 2

In the compound [Lu(C_7_H_7_)_3_(C_4_H_8_O)_2_], the Lu ion is coordinated by three benzyl and two tetra­hydro­furan ligands. Two of the benzyl groups are bonded in a classical η^1^-fashion through the methyl­ene *via* the *ipso*-carbon atom of the benzyl ligand in addition to bonding through the methyl­ene C atom, resulting in a modified trigonal–bipyramidal coordination geometry about the Lu center.

## Chemical context   

The chemistry of σ-bonded rare-earth metal (RE) hydro­carbyl complexes has a long and rich history (Zimmermann & Anwander, 2010 ▸), with the compounds being versatile synthetic precursors and involved in important polymerization and various catalytic transformations. Lappert & Pearce (1973 ▸) reported the synthesis of the first well-defined homoleptic trialkyl compounds utilizing neopentyl and tri­methyl­silylmethyl ligands, [*RE*(CH_2_^*t*^Bu)_3_(THF)_2_] and [*RE*(CH_2_SiMe_3_)_3_(THF)_2_] (*RE* = Sc, Y). More recently, the benzyl ligand (CH_2_Ph) has been successfully employed to provide access to a wide range of [*RE*(CH_2_Ph)_3_(THF)_*x*_] (*x* = 2, 3) compounds (Bambirra *et al.*, 2006 ▸; Döring & Kempe, 2008 ▸; Meyer *et al.*, 2008 ▸; Wooles *et al.*, 2010 ▸; Huang *et al.*, 2013 ▸). The bonding between the rare-earth metal and benzyl ligands depends both on the size of metal and the number of coord­inated THF ligands. In the series of tris-THF derivatives [*RE*(CH_2_Ph)_3_(THF)_3_], in line with the lanthanide contraction, the bonding changes from three η^2^-bonded benzyl ligands for the large early, to a mix of η^1^-/η^2^-benzyls for the mid- and three η^1^-benzyls for the smaller, late metals (Wooles *et al.*, 2010 ▸). Metal size also matters for bis-THF compounds, [*RE*(CH_2_Ph)_3_(THF)_2_]; the small scandium atom can only support three η^1^-bound benzyls (Meyer *et al.*, 2008 ▸) whereas [Er(CH_2_Ph)_3_(THF)_2_] features one η^2^- and two η^1^-coordinated benzyl ligands (Huang *et al.*, 2013 ▸). Here we report the solid-state X-ray structure of [Lu(CH_2_Ph)_3_(THF)_2_].

## Structural commentary   

**Figure 1:**
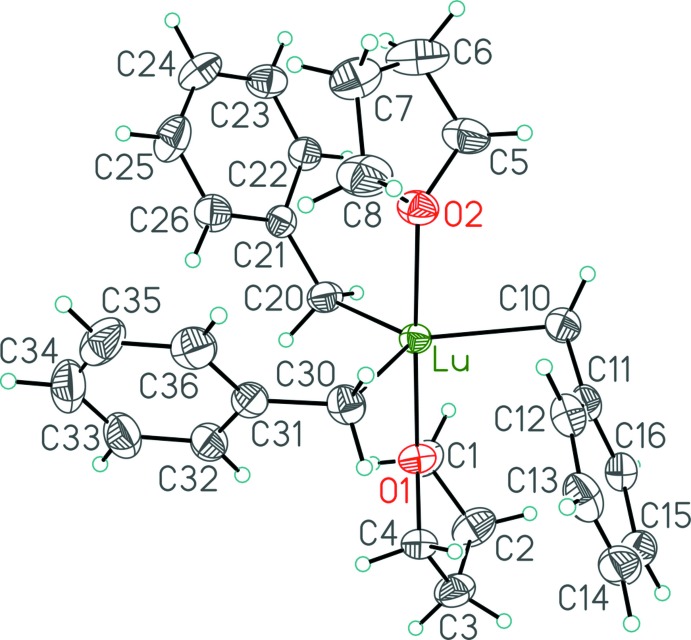
Mol­ecular structure of **1** in the crystal. Displacement ellipsoids are shown at the 50% probability level. Hydrogen atoms are shown with arbitrarily small displacement parameters.

The mol­ecular structure of [Lu(CH_2_Ph)_3_(THF)_2_] (**1**) (Fig. 1 ▸) reveals that the Lu center is coordinated by two oxygen atoms of the THF ligands and three methyl­ene carbon atoms of the benzyl groups. The disposition of the two THF ligands about the lutetium center is almost linear [O1—Lu—O2 = 177.10 (6)°], thus suggesting a trigonal–bipyramidal structure with the two THF ligands occupying the axial sites and the benzyl groups in the equatorial positions, consistent with the observed solution behavior (Meyer *et al.*, 2008 ▸). The Lu—C distances are essentially equal [Lu—C10 = 2.401 (3), Lu—C20 = 2.380 (3), Lu—C30 = 2.404 (3) Å] and the equatorial C—Lu—C angles are close to the expected value of 120° [C10—Lu—C20 = 121.59 (10), C10—Lu—C30 = 123.98 (9), C20—Lu—C30 = 114.38 (10)°], albeit with some deviation from the ideal value. This deviation can be attributed to the presence of an additional inter­action from the *ipso* carbon atom of one of the benzyl ligands, as reflected in the Lu—C_*ipso*_ distances and Lu—C—C_*ipso*_ angles: Lu—C11 = 2.920 (3) *vs* 3.317 (2) and 3.267 (3) Å, for Lu—C21 and Lu—C31, respectively, and Lu—C10—C11 = 94.94 (16) *vs* Lu—C20—C21 116.79 (17) and Lu—C30—C31 112.80 (17)°. At the same time, the bond distance between the benzylic and *ipso* carbon atoms for the η^2^-bonded benzyl group [C10—C11 = 1.467 (4) Å] is not significantly different from those of the η^1^-bonded benzyls [C20—C21 = 1.475 (3), C30—C31 = 1.470 (4) Å].
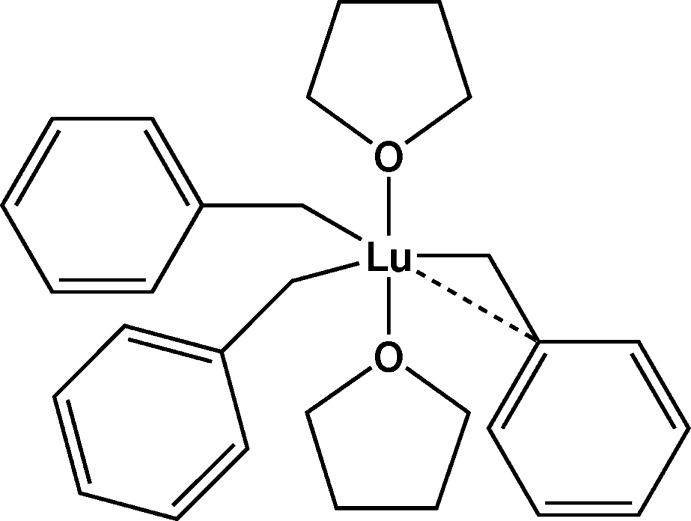


The mixed modes of benzyl coordination in the title compound are in contrast to the structure of the related hexa­coordinate tris-THF compound, [Lu(CH_2_Ph)_3_(THF)_3_], in which all of the benzyl ligands are η^1^-coordinated (Meyer *et al.*, 2008 ▸, 2013 ▸). The structural results provide yet another example of the importance of the metal size in the series of homologous [*RE*(CH_2_Ph)_3_(THF)_2_] (*RE* = Sc, Er, Lu) compounds: the complex featuring the small scandium center shows all three benzyl ligands adopting the η^1^-bonding mode (Meyer *et al.*, 2008 ▸), whereas the larger lutetium can allow one of the three benzyl ligands to adopt the more sterically-demanding η^2^-bonding mode; indeed, the Lu compound is isomorphous with the similarly-sized erbium complex, [Er(η^2^-CH_2_Ph)(η^1^-CH_2_Ph)_2_(THF)_2_] (Huang *et al.*, 2013 ▸), with metrical parameters reflecting the small decrease in ionic radius from erbium to lutetium (Shannon, 1976 ▸).

## Supra­molecular features   

The closest inter­molecular contacts are between benzyl carbons C11 and C12 and the THF methyl­ene-group hydrogen H1*B* (at *x* − 1, *y*, *z*), at 2.80 and 2.89 Å, respectively, and between the benzyl carbon C16 and the phenyl-group hydrogen H22 (at −*x*, −*y*, 1 − *z*), at 2.86 Å. These interactions connect the complexes in a supramolecular ribbon running along the *a*-axis direction

## Database survey   

For related lanthanide complexes of the form [*M*(CH_2_Ph)_3_(THF)_2_], only the structure of the Er analogue has been reported (Huang *et al.*, 2013 ▸); the structure of the related Sc complex has also been described (Meyer *et al.*, 2008 ▸). The structures of the [M(CH_2_Ph)_3_(THF)_3_] complexes have been more exhaustively determined, with the lanthanides La (Bambirra *et al.*, 2006 ▸), Ce (Wooles *et al.*, 2010 ▸), Pr (Wooles *et al.*, 2010 ▸), Nd (Döring & Kempe, 2008 ▸; Wooles *et al.*, 2010 ▸), Sm (Wooles *et al.*, 2010 ▸), Gd (Wooles *et al.*, 2010 ▸; Huang *et al.*, 2013 ▸), Dy (Wooles *et al.*, 2010 ▸), Ho (Huang *et al.*, 2013 ▸), Er (Wooles *et al.*, 2010 ▸; Huang *et al.*, 2013 ▸), and Lu (Meyer *et al.*, 2008 ▸) being reported, the related Sc (Meyer *et al.*, 2008 ▸) and Y (Hardera *et al.*, 2008 ▸; Mills *et al.* 2009 ▸) analogues are also known.

## Synthesis and crystallization   

The synthesis, solution structure and spectroscopic characterization of [Lu(CH_2_Ph)_3_(THF)_2_] (**1**) have been reported previously (Meyer *et al.*, 2008 ▸). The preparation and characterization of the related compounds [Sc(CH_2_Ph)_3_(THF)_2_] and [*RE*(CH_2_Ph)_3_(THF)_2_] (*RE* = Sc, Lu) were also reported at that time.

X-ray quality crystals of compound **1** were obtained by cooling a dilute toluene solution of the compound to 243 K for several days.

## Refinement   

Crystal data, data collection and structure refinement details are summarized in Table 1 ▸. Hydrogen atoms were generated in idealized positions according to the *sp* ^2^ or *sp* ^3^ geometries of their attached carbon atoms, and given isotropic displacement parameters *U*_iso_(H) = 1.2*U*_eq_(parent atom). C—H distances in the CH_2_ groups were constrained to 0.99 Å and those in phenyl-ring C–H groups to 0.95 Å.

## Supplementary Material

Crystal structure: contains datablock(s) I, New_Global_Publ_Block. DOI: 10.1107/S2056989017018254/pj2048sup1.cif

Structure factors: contains datablock(s) I. DOI: 10.1107/S2056989017018254/pj2048Isup2.hkl

CCDC reference: 1812810

Additional supporting information:  crystallographic information; 3D view; checkCIF report

## Figures and Tables

**Table 1:** Experimental details

Crystal data	
Chemical formula	[Lu(C_7_H_7_)_3_(C_4_H_8_O)_2_]
*M*_r_	592.55
Crystal system, space group	Triclinic, *P* 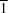
Temperature (K)	193
*a*, *b*, *c* (Å)	7.7103 (7), 12.7416 (11), 14.2187 (12)
α, β, γ (°)	75.1572 (11), 77.8324 (11), 73.4904 (11)
*V* (Å^3^)	1280.16 (19)
*Z*	2
Radiation type	Mo *K*α
μ (mm^−1^)	3.88
Crystal size (mm)	0.48 × 0.10 × 0.09

Data collection	
Diffractometer	Bruker SMART 1000 CCD detector/PLATFORM
Absorption correction	Numerical (*SADABS*; Bruker, 2015 ▸)
*T*_min_, *T*_max_	0.216, 0.764
No. of measured, independent and observed [*I* > 2σ(*I*)] reflections	11301, 5803, 5331
*R*_int_	0.020
(sin θ/λ)_max_ (Å^−1^)	0.649

Refinement	
*R*[*F* ^2^ > 2σ(*F* ^2^)], *wR*(*F* ^2^), *S*	0.021, 0.051, 1.07
No. of reflections	5803
No. of parameters	289
H-atom treatment	H-atom parameters constrained
Δρ_max_, Δρ_min_ (e Å^−3^)	1.00, −0.36

Computer programs: *SMART* and *SAINT* (Bruker, 2008 ▸), *SHELXS97* and *SHELXTL* (Sheldrick, 2008 ▸) and *SHELXL2014* (Sheldrick, 2015 ▸).

## supplementary crystallographic information

### Crystal data

**Table:**

[Lu(C_7_H_7_)_3_(C_4_H_8_O)_2_]	*Z* = 2
*M**_r_* = 592.55	*F*(000) = 596
Triclinic, *P*1	*D*_x_ = 1.537 Mg m^−^^3^
*a* = 7.7103 (7) Å	Mo *K*α radiation, λ = 0.71073 Å
*b* = 12.7416 (11) Å	Cell parameters from 5362 reflections
*c* = 14.2187 (12) Å	θ = 2.8–27.4°
α = 75.1572 (11)°	µ = 3.88 mm^−^^1^
β = 77.8324 (11)°	*T* = 193 K
γ = 73.4904 (11)°	Prism, colorless
*V* = 1280.16 (19) Å^3^	0.48 × 0.10 × 0.09 mm

### Data collection

**Table:**

Bruker SMART 1000 CCD detector/PLATFORM diffractometer	5331 reflections with *I* > 2σ(*I*)
ω scans	*R*_int_ = 0.020
Absorption correction: numerical (SADABS; Bruker, 2015)	θ_max_ = 27.5°, θ_min_ = 1.7°
*T*_min_ = 0.216, *T*_max_ = 0.764	*h* = −10→10
11301 measured reflections	*k* = −16→16
5803 independent reflections	*l* = −18→18

### Refinement

**Table:**

Refinement on *F*^2^	0 restraints
Least-squares matrix: full	Hydrogen site location: inferred from neighbouring sites
*R*[*F*^2^ > 2σ(*F*^2^)] = 0.021	H-atom parameters constrained
*wR*(*F*^2^) = 0.051	*w* = 1/[σ^2^(*F*_o_^2^) + (0.0273*P*)^2^] where *P* = (*F*_o_^2^ + 2*F*_c_^2^)/3
*S* = 1.07	(Δ/σ)_max_ = 0.001
5803 reflections	Δρ_max_ = 1.00 e Å^−^^3^
289 parameters	Δρ_min_ = −0.36 e Å^−^^3^

### Special details

**Table:**

Geometry. All esds (except the esd in the dihedral angle between two l.s. planes) are estimated using the full covariance matrix. The cell esds are taken into account individually in the estimation of esds in distances, angles and torsion angles; correlations between esds in cell parameters are only used when they are defined by crystal symmetry. An approximate (isotropic) treatment of cell esds is used for estimating esds involving l.s. planes.

### Fractional atomic coordinates and isotropic or equivalent isotropic displacement parameters (Å^2^)

**Table:**

	*x*	*y*	*z*	*U*_iso_*/*U*_eq_	
Lu	0.13939 (2)	0.09124 (2)	0.27792 (2)	0.02548 (4)	
O1	0.3538 (2)	−0.07199 (15)	0.26404 (13)	0.0309 (4)	
O2	−0.0789 (3)	0.25126 (16)	0.30019 (14)	0.0364 (4)	
C1	0.4592 (4)	−0.1372 (2)	0.3424 (2)	0.0389 (6)	
H1A	0.3942	−0.1209	0.4069	0.047*	
H1B	0.5808	−0.1205	0.3305	0.047*	
C2	0.4779 (5)	−0.2566 (2)	0.3395 (2)	0.0497 (8)	
H2A	0.3715	−0.2836	0.3798	0.060*	
H2B	0.5910	−0.3054	0.3644	0.060*	
C3	0.4862 (4)	−0.2552 (2)	0.2311 (2)	0.0434 (7)	
H3A	0.6144	−0.2781	0.1995	0.052*	
H3B	0.4172	−0.3066	0.2235	0.052*	
C4	0.3991 (4)	−0.1345 (2)	0.1856 (2)	0.0349 (6)	
H4A	0.4857	−0.1035	0.1309	0.042*	
H4B	0.2874	−0.1304	0.1596	0.042*	
C5	−0.1912 (4)	0.2700 (3)	0.3927 (2)	0.0477 (8)	
H5A	−0.1255	0.2260	0.4485	0.057*	
H5B	−0.3066	0.2471	0.4005	0.057*	
C6	−0.2297 (6)	0.3907 (3)	0.3903 (3)	0.0657 (11)	
H6A	−0.1479	0.4053	0.4280	0.079*	
H6B	−0.3581	0.4191	0.4190	0.079*	
C7	−0.1960 (6)	0.4459 (3)	0.2846 (3)	0.0719 (12)	
H7A	−0.3117	0.4942	0.2625	0.086*	
H7B	−0.1083	0.4929	0.2746	0.086*	
C8	−0.1194 (5)	0.3550 (3)	0.2286 (3)	0.0578 (9)	
H8A	−0.2094	0.3528	0.1891	0.069*	
H8B	−0.0068	0.3673	0.1836	0.069*	
C10	−0.0726 (4)	−0.0116 (2)	0.3812 (2)	0.0362 (6)	
H10A	−0.0231	−0.0627	0.4398	0.043*	
H10B	−0.1927	0.0375	0.4011	0.043*	
C11	−0.0786 (3)	−0.0711 (2)	0.30660 (19)	0.0303 (5)	
C12	−0.1372 (3)	−0.0116 (2)	0.2159 (2)	0.0352 (6)	
H12	−0.1848	0.0672	0.2063	0.042*	
C13	−0.1277 (4)	−0.0643 (3)	0.1403 (2)	0.0423 (7)	
H13	−0.1649	−0.0211	0.0795	0.051*	
C14	−0.0648 (4)	−0.1785 (3)	0.1527 (2)	0.0442 (7)	
H14	−0.0597	−0.2149	0.1012	0.053*	
C15	−0.0090 (4)	−0.2399 (3)	0.2413 (2)	0.0405 (6)	
H15	0.0331	−0.3190	0.2509	0.049*	
C16	−0.0139 (3)	−0.1875 (2)	0.3159 (2)	0.0327 (6)	
H16	0.0278	−0.2315	0.3754	0.039*	
C20	0.3638 (4)	0.1353 (2)	0.3431 (2)	0.0343 (6)	
H20A	0.3766	0.0859	0.4087	0.041*	
H20B	0.4825	0.1169	0.3002	0.041*	
C21	0.3296 (3)	0.2520 (2)	0.35345 (18)	0.0284 (5)	
C22	0.2680 (4)	0.2820 (2)	0.44517 (19)	0.0323 (5)	
H22	0.2537	0.2251	0.5022	0.039*	
C23	0.2274 (4)	0.3919 (2)	0.4553 (2)	0.0406 (7)	
H23	0.1844	0.4092	0.5186	0.049*	
C24	0.2491 (4)	0.4766 (2)	0.3739 (2)	0.0466 (7)	
H24	0.2219	0.5522	0.3806	0.056*	
C25	0.3113 (5)	0.4489 (3)	0.2824 (2)	0.0483 (8)	
H25	0.3271	0.5061	0.2258	0.058*	
C26	0.3506 (4)	0.3393 (3)	0.2724 (2)	0.0396 (6)	
H26	0.3931	0.3227	0.2089	0.048*	
C30	0.1539 (4)	0.1513 (2)	0.10243 (19)	0.0347 (6)	
H30A	0.1860	0.0846	0.0728	0.042*	
H30B	0.0316	0.1961	0.0870	0.042*	
C31	0.2885 (4)	0.2184 (2)	0.05797 (18)	0.0327 (6)	
C32	0.4758 (4)	0.1712 (2)	0.0604 (2)	0.0385 (6)	
H32	0.5154	0.0938	0.0884	0.046*	
C33	0.6049 (5)	0.2338 (3)	0.0233 (2)	0.0494 (8)	
H33	0.7307	0.1985	0.0256	0.059*	
C34	0.5541 (6)	0.3459 (3)	−0.0166 (2)	0.0586 (10)	
H34	0.6428	0.3888	−0.0409	0.070*	
C35	0.3700 (6)	0.3952 (3)	−0.0209 (2)	0.0580 (10)	
H35	0.3329	0.4727	−0.0489	0.070*	
C36	0.2393 (5)	0.3337 (2)	0.0149 (2)	0.0441 (7)	
H36	0.1144	0.3696	0.0104	0.053*	

### Atomic displacement parameters (Å^2^)

**Table:**

	*U*^11^	*U*^22^	*U*^33^	*U*^12^	*U*^13^	*U*^23^
Lu	0.02754 (6)	0.02497 (6)	0.02355 (6)	−0.00524 (4)	−0.00317 (4)	−0.00625 (4)
O1	0.0330 (9)	0.0288 (9)	0.0321 (9)	−0.0021 (7)	−0.0084 (7)	−0.0117 (8)
O2	0.0355 (10)	0.0323 (10)	0.0362 (10)	−0.0003 (8)	−0.0041 (8)	−0.0079 (8)
C1	0.0440 (15)	0.0323 (14)	0.0418 (16)	−0.0057 (12)	−0.0175 (12)	−0.0051 (12)
C2	0.064 (2)	0.0316 (16)	0.054 (2)	−0.0066 (14)	−0.0202 (16)	−0.0051 (14)
C3	0.0460 (16)	0.0315 (15)	0.0531 (19)	−0.0040 (13)	−0.0076 (14)	−0.0154 (13)
C4	0.0382 (14)	0.0343 (14)	0.0339 (14)	−0.0088 (11)	0.0003 (11)	−0.0148 (12)
C5	0.0514 (18)	0.0472 (18)	0.0407 (17)	−0.0029 (14)	−0.0001 (13)	−0.0182 (14)
C6	0.075 (3)	0.049 (2)	0.069 (2)	0.0114 (18)	−0.017 (2)	−0.0295 (19)
C7	0.074 (3)	0.0370 (19)	0.094 (3)	−0.0066 (18)	0.007 (2)	−0.019 (2)
C8	0.069 (2)	0.0347 (18)	0.052 (2)	0.0083 (15)	−0.0066 (17)	−0.0025 (14)
C10	0.0388 (14)	0.0396 (15)	0.0318 (14)	−0.0131 (12)	0.0000 (11)	−0.0109 (12)
C11	0.0237 (12)	0.0372 (14)	0.0306 (13)	−0.0116 (10)	0.0004 (9)	−0.0068 (11)
C12	0.0287 (13)	0.0381 (15)	0.0346 (14)	−0.0088 (11)	−0.0040 (10)	0.0002 (12)
C13	0.0316 (14)	0.062 (2)	0.0317 (14)	−0.0145 (13)	−0.0062 (11)	−0.0035 (13)
C14	0.0427 (16)	0.060 (2)	0.0379 (16)	−0.0173 (15)	−0.0035 (12)	−0.0213 (14)
C15	0.0346 (14)	0.0392 (16)	0.0513 (18)	−0.0105 (12)	−0.0042 (12)	−0.0158 (13)
C16	0.0316 (13)	0.0352 (14)	0.0320 (14)	−0.0126 (11)	−0.0049 (10)	−0.0031 (11)
C20	0.0338 (13)	0.0333 (14)	0.0384 (15)	−0.0063 (11)	−0.0051 (11)	−0.0143 (12)
C21	0.0242 (11)	0.0346 (14)	0.0311 (13)	−0.0104 (10)	−0.0052 (9)	−0.0105 (11)
C22	0.0356 (13)	0.0349 (14)	0.0279 (13)	−0.0104 (11)	−0.0051 (10)	−0.0069 (11)
C23	0.0447 (16)	0.0444 (17)	0.0376 (15)	−0.0083 (13)	−0.0085 (12)	−0.0183 (13)
C24	0.061 (2)	0.0310 (15)	0.0554 (19)	−0.0121 (14)	−0.0175 (15)	−0.0138 (14)
C25	0.068 (2)	0.0383 (16)	0.0426 (17)	−0.0247 (15)	−0.0144 (15)	0.0032 (13)
C26	0.0464 (16)	0.0466 (17)	0.0308 (14)	−0.0174 (13)	−0.0027 (12)	−0.0126 (12)
C30	0.0377 (14)	0.0403 (15)	0.0262 (13)	−0.0108 (12)	−0.0058 (10)	−0.0049 (11)
C31	0.0468 (15)	0.0342 (14)	0.0187 (11)	−0.0139 (12)	−0.0034 (10)	−0.0051 (10)
C32	0.0454 (16)	0.0413 (16)	0.0298 (14)	−0.0151 (13)	−0.0012 (11)	−0.0078 (12)
C33	0.0524 (18)	0.067 (2)	0.0346 (16)	−0.0300 (16)	0.0032 (13)	−0.0123 (15)
C34	0.084 (3)	0.074 (3)	0.0341 (17)	−0.055 (2)	0.0032 (16)	−0.0087 (16)
C35	0.109 (3)	0.0387 (17)	0.0314 (16)	−0.0331 (19)	−0.0090 (17)	−0.0009 (13)
C36	0.0621 (19)	0.0396 (16)	0.0268 (14)	−0.0100 (14)	−0.0068 (13)	−0.0030 (12)

### Geometric parameters (Å, º)

**Table:**

Lu—O1	2.2839 (17)	C12—C13	1.385 (4)
Lu—O2	2.2902 (18)	C12—H12	0.9500
Lu—C20	2.380 (3)	C13—C14	1.375 (5)
Lu—C10	2.401 (3)	C13—H13	0.9500
Lu—C30	2.404 (3)	C14—C15	1.385 (4)
Lu—C11	2.920 (3)	C14—H14	0.9500
O1—C1	1.455 (3)	C15—C16	1.379 (4)
O1—C4	1.461 (3)	C15—H15	0.9500
O2—C8	1.446 (4)	C16—H16	0.9500
O2—C5	1.450 (3)	C20—C21	1.475 (3)
C1—C2	1.498 (4)	C20—H20A	0.9900
C1—H1A	0.9900	C20—H20B	0.9900
C1—H1B	0.9900	C21—C26	1.399 (4)
C2—C3	1.526 (4)	C21—C22	1.401 (3)
C2—H2A	0.9900	C22—C23	1.383 (4)
C2—H2B	0.9900	C22—H22	0.9500
C3—C4	1.523 (4)	C23—C24	1.384 (4)
C3—H3A	0.9900	C23—H23	0.9500
C3—H3B	0.9900	C24—C25	1.387 (4)
C4—H4A	0.9900	C24—H24	0.9500
C4—H4B	0.9900	C25—C26	1.380 (4)
C5—C6	1.474 (4)	C25—H25	0.9500
C5—H5A	0.9900	C26—H26	0.9500
C5—H5B	0.9900	C30—C31	1.470 (4)
C6—C7	1.489 (5)	C30—H30A	0.9900
C6—H6A	0.9900	C30—H30B	0.9900
C6—H6B	0.9900	C31—C32	1.402 (4)
C7—C8	1.488 (5)	C31—C36	1.413 (4)
C7—H7A	0.9900	C32—C33	1.386 (4)
C7—H7B	0.9900	C32—H32	0.9500
C8—H8A	0.9900	C33—C34	1.369 (5)
C8—H8B	0.9900	C33—H33	0.9500
C10—C11	1.467 (4)	C34—C35	1.386 (5)
C10—H10A	0.9900	C34—H34	0.9500
C10—H10B	0.9900	C35—C36	1.384 (5)
C11—C16	1.405 (4)	C35—H35	0.9500
C11—C12	1.412 (4)	C36—H36	0.9500
			
O1—Lu—O2	177.10 (6)	C11—C10—H10B	112.7
O1—Lu—C20	84.95 (8)	Lu—C10—H10B	112.7
O2—Lu—C20	94.44 (8)	H10A—C10—H10B	110.2
O1—Lu—C10	90.54 (8)	C16—C11—C12	115.5 (2)
O2—Lu—C10	87.36 (8)	C16—C11—C10	123.5 (2)
C20—Lu—C10	121.59 (10)	C12—C11—C10	120.8 (3)
O1—Lu—C30	92.12 (8)	C16—C11—Lu	127.02 (17)
O2—Lu—C30	90.72 (8)	C12—C11—Lu	86.53 (16)
C20—Lu—C30	114.38 (10)	C10—C11—Lu	55.02 (13)
C10—Lu—C30	123.98 (9)	C13—C12—C11	122.2 (3)
O1—Lu—C11	76.43 (7)	C13—C12—H12	118.9
O2—Lu—C11	102.58 (7)	C11—C12—H12	118.9
C20—Lu—C11	143.56 (9)	C14—C13—C12	120.4 (3)
C10—Lu—C11	30.04 (8)	C14—C13—H13	119.8
C30—Lu—C11	97.55 (8)	C12—C13—H13	119.8
C1—O1—C4	108.2 (2)	C13—C14—C15	119.0 (3)
C1—O1—Lu	122.45 (15)	C13—C14—H14	120.5
C4—O1—Lu	129.13 (16)	C15—C14—H14	120.5
C8—O2—C5	107.1 (2)	C16—C15—C14	120.8 (3)
C8—O2—Lu	127.16 (18)	C16—C15—H15	119.6
C5—O2—Lu	125.49 (17)	C14—C15—H15	119.6
O1—C1—C2	104.6 (2)	C15—C16—C11	122.0 (3)
O1—C1—H1A	110.8	C15—C16—H16	119.0
C2—C1—H1A	110.8	C11—C16—H16	119.0
O1—C1—H1B	110.8	C21—C20—Lu	116.79 (17)
C2—C1—H1B	110.8	C21—C20—H20A	108.1
H1A—C1—H1B	108.9	Lu—C20—H20A	108.1
C1—C2—C3	104.5 (2)	C21—C20—H20B	108.1
C1—C2—H2A	110.9	Lu—C20—H20B	108.1
C3—C2—H2A	110.8	H20A—C20—H20B	107.3
C1—C2—H2B	110.8	C26—C21—C22	116.3 (2)
C3—C2—H2B	110.9	C26—C21—C20	122.2 (2)
H2A—C2—H2B	108.9	C22—C21—C20	121.5 (2)
C4—C3—C2	105.1 (2)	C23—C22—C21	122.1 (3)
C4—C3—H3A	110.7	C23—C22—H22	118.9
C2—C3—H3A	110.7	C21—C22—H22	118.9
C4—C3—H3B	110.7	C22—C23—C24	120.4 (3)
C2—C3—H3B	110.7	C22—C23—H23	119.8
H3A—C3—H3B	108.8	C24—C23—H23	119.8
O1—C4—C3	106.5 (2)	C23—C24—C25	118.5 (3)
O1—C4—H4A	110.4	C23—C24—H24	120.7
C3—C4—H4A	110.4	C25—C24—H24	120.7
O1—C4—H4B	110.4	C26—C25—C24	120.9 (3)
C3—C4—H4B	110.4	C26—C25—H25	119.5
H4A—C4—H4B	108.6	C24—C25—H25	119.5
O2—C5—C6	106.9 (3)	C25—C26—C21	121.7 (3)
O2—C5—H5A	110.3	C25—C26—H26	119.1
C6—C5—H5A	110.3	C21—C26—H26	119.1
O2—C5—H5B	110.3	C31—C30—Lu	112.80 (17)
C6—C5—H5B	110.3	C31—C30—H30A	109.0
H5A—C5—H5B	108.6	Lu—C30—H30A	109.0
C5—C6—C7	106.0 (3)	C31—C30—H30B	109.0
C5—C6—H6A	110.5	Lu—C30—H30B	109.0
C7—C6—H6A	110.5	H30A—C30—H30B	107.8
C5—C6—H6B	110.5	C32—C31—C36	115.9 (3)
C7—C6—H6B	110.5	C32—C31—C30	120.8 (2)
H6A—C6—H6B	108.7	C36—C31—C30	123.2 (3)
C8—C7—C6	106.8 (3)	C33—C32—C31	122.1 (3)
C8—C7—H7A	110.4	C33—C32—H32	119.0
C6—C7—H7A	110.4	C31—C32—H32	119.0
C8—C7—H7B	110.4	C34—C33—C32	121.0 (3)
C6—C7—H7B	110.4	C34—C33—H33	119.5
H7A—C7—H7B	108.6	C32—C33—H33	119.5
O2—C8—C7	106.8 (3)	C33—C34—C35	118.4 (3)
O2—C8—H8A	110.4	C33—C34—H34	120.8
C7—C8—H8A	110.4	C35—C34—H34	120.8
O2—C8—H8B	110.4	C36—C35—C34	121.3 (3)
C7—C8—H8B	110.4	C36—C35—H35	119.3
H8A—C8—H8B	108.6	C34—C35—H35	119.3
C11—C10—Lu	94.94 (16)	C35—C36—C31	121.2 (3)
C11—C10—H10A	112.7	C35—C36—H36	119.4
Lu—C10—H10A	112.7	C31—C36—H36	119.4
			
O2—Lu—O1—C1	−33.1 (13)	C20—Lu—C11—C12	−173.55 (15)
C20—Lu—O1—C1	45.0 (2)	C10—Lu—C11—C12	131.9 (2)
C10—Lu—O1—C1	−76.7 (2)	C30—Lu—C11—C12	−21.82 (17)
C30—Lu—O1—C1	159.2 (2)	O1—Lu—C11—C10	115.88 (17)
C11—Lu—O1—C1	−103.5 (2)	O2—Lu—C11—C10	−61.32 (17)
O2—Lu—O1—C4	140.7 (11)	C20—Lu—C11—C10	54.5 (2)
C20—Lu—O1—C4	−141.2 (2)	C30—Lu—C11—C10	−153.75 (17)
C10—Lu—O1—C4	97.1 (2)	C16—C11—C12—C13	−1.4 (4)
C30—Lu—O1—C4	−26.9 (2)	C10—C11—C12—C13	173.6 (2)
C11—Lu—O1—C4	70.3 (2)	Lu—C11—C12—C13	128.4 (2)
O1—Lu—O2—C8	173.0 (11)	C11—C12—C13—C14	2.0 (4)
C20—Lu—O2—C8	95.2 (3)	C12—C13—C14—C15	−0.8 (4)
C10—Lu—O2—C8	−143.3 (3)	C13—C14—C15—C16	−0.8 (4)
C30—Lu—O2—C8	−19.3 (3)	C14—C15—C16—C11	1.4 (4)
C11—Lu—O2—C8	−117.2 (3)	C12—C11—C16—C15	−0.3 (4)
O1—Lu—O2—C5	−0.9 (13)	C10—C11—C16—C15	−175.1 (3)
C20—Lu—O2—C5	−78.7 (2)	Lu—C11—C16—C15	−106.3 (3)
C10—Lu—O2—C5	42.7 (2)	O1—Lu—C20—C21	167.8 (2)
C30—Lu—O2—C5	166.7 (2)	O2—Lu—C20—C21	−15.0 (2)
C11—Lu—O2—C5	68.8 (2)	C10—Lu—C20—C21	−104.7 (2)
C4—O1—C1—C2	−32.1 (3)	C30—Lu—C20—C21	77.8 (2)
Lu—O1—C1—C2	142.9 (2)	C11—Lu—C20—C21	−133.25 (18)
O1—C1—C2—C3	32.7 (3)	Lu—C20—C21—C26	−74.3 (3)
C1—C2—C3—C4	−21.5 (3)	Lu—C20—C21—C22	103.4 (2)
C1—O1—C4—C3	18.3 (3)	C26—C21—C22—C23	0.9 (4)
Lu—O1—C4—C3	−156.28 (18)	C20—C21—C22—C23	−177.0 (2)
C2—C3—C4—O1	2.6 (3)	C21—C22—C23—C24	−0.8 (4)
C8—O2—C5—C6	−26.3 (4)	C22—C23—C24—C25	0.3 (5)
Lu—O2—C5—C6	148.7 (2)	C23—C24—C25—C26	0.1 (5)
O2—C5—C6—C7	19.6 (4)	C24—C25—C26—C21	0.0 (5)
C5—C6—C7—C8	−5.9 (5)	C22—C21—C26—C25	−0.5 (4)
C5—O2—C8—C7	22.3 (4)	C20—C21—C26—C25	177.4 (3)
Lu—O2—C8—C7	−152.5 (2)	O1—Lu—C30—C31	−91.35 (19)
C6—C7—C8—O2	−9.9 (5)	O2—Lu—C30—C31	89.27 (19)
O1—Lu—C10—C11	−61.00 (16)	C20—Lu—C30—C31	−5.9 (2)
O2—Lu—C10—C11	121.00 (17)	C10—Lu—C30—C31	176.57 (17)
C20—Lu—C10—C11	−145.40 (15)	C11—Lu—C30—C31	−167.94 (19)
C30—Lu—C10—C11	31.9 (2)	Lu—C30—C31—C32	65.0 (3)
Lu—C10—C11—C16	114.7 (2)	Lu—C30—C31—C36	−112.0 (2)
Lu—C10—C11—C12	−59.8 (2)	C36—C31—C32—C33	0.3 (4)
O1—Lu—C11—C16	7.5 (2)	C30—C31—C32—C33	−176.8 (3)
O2—Lu—C11—C16	−169.7 (2)	C31—C32—C33—C34	0.8 (5)
C20—Lu—C11—C16	−53.9 (3)	C32—C33—C34—C35	−1.2 (5)
C10—Lu—C11—C16	−108.4 (3)	C33—C34—C35—C36	0.5 (5)
C30—Lu—C11—C16	97.9 (2)	C34—C35—C36—C31	0.6 (5)
O1—Lu—C11—C12	−112.19 (16)	C32—C31—C36—C35	−1.0 (4)
O2—Lu—C11—C12	70.62 (16)	C30—C31—C36—C35	176.1 (3)
